# Reversal of Prediabetes in Saudi Adults: Results from an 18 Month Lifestyle Intervention

**DOI:** 10.3390/nu12030804

**Published:** 2020-03-18

**Authors:** Osama E. Amer, Shaun Sabico, Hanan A. Alfawaz, Naji Aljohani, Syed Danish Hussain, Abdullah M. Alnaami, Kaiser Wani, Nasser M. Al-Daghri

**Affiliations:** 1 Chair for Biomarkers of Chronic Diseases, College of Science, King Saud University, Riyadh 11451, Saudi Arabia; osamaemam@gmail.com (O.E.A.); eaglescout01@yahoo.com (S.S.); halfawaz@ksu.edu.sa (H.A.A.); danishhussain121@gmail.com (S.D.H.); aalnaami@yahoo.com (A.M.A.); wani.kaiser@gmail.com (K.W.); 2 Biochemistry Department, College of Science, King Saud University, Riyadh 11451, Saudi Arabia; 3 Department of Food Science and Nutrition, College of Food Science & Agriculture, King Saud University, Riyadh 11451, Saudi Arabia; 4 Specialized Diabetes and Endocrine Center, King Fahad Medical City, Riyadh 11525, Saudi Arabia; najij@hotmail.com

**Keywords:** prediabetes, lifestyle interventions, Saudi, prevention, type 2 diabetes

## Abstract

Aim: This 18 month intervention study aims to determine the efficacy of a lifestyle modification program on prediabetes reversal among Saudi adults. Methods: An 18 month randomized, multicenter trial was conducted among Saudis with prediabetes aged 25–60 recruited from King Salman Hospital and primary care centers in Riyadh, Saudi Arabia. A total of 180 consenting individuals were randomized (1:1) to receive either intensive lifestyle intervention (ILIG) or guidance (control group, CG). ILIG was provided with a personalized lifestyle counseling by nutritionists every 3 months to improve diet and exercise behaviors. CG was given booklets containing information on prediabetes and its prevention. Data from lifestyle assessments and laboratory measurements were analyzed at baseline and every 6 months. The primary outcome was the reversal rate of prediabetes. Results: 158 participants were analyzed (CG:85, ILIG:73) at the 12 month follow-up and 28 participants (CG:11 and ILIG:17) completed the entire 18 month study. Post-intervention, the cumulative incidence of prediabetes reversal in the ILIG was 38 participants (52.1%) which was significantly higher than CG with 26 participants (30.6%) (*p* = 0.02). Conclusion: A tailored lifestyle intervention is effective in reversing prediabetes, at least for a year, among Arab adults with prediabetes. The challenge of sustaining interest in adopting lifestyle changes for a longer duration should be addressed in this population.

## 1. Introduction

In 2019, the International Diabetes Federation (IFG) stated that an estimated 7.5% (374 million) of people aged 20–79 years worldwide are living with prediabetes [1]. It has also been observed that individuals with untreated prediabetes have a 5%–10% chance of developing type 2 diabetes mellitus (T2DM), within a year [2]. In addition, these subjects have a higher risk of cardiovascular disorders and comorbidities [3,4]. According to the World Health Organization (WHO) [5], Saudi Arabia is seventh globally and second in the Middle East region, with the highest prevalence of T2DM. The rapid industrial development in Saudi Arabia led to a remarkable increase in the standards of living, consequently adopting a more Western lifestyle, which resulted in unhealthy dietary patterns and low levels of physical activity, factors that led to the increase in prevalence of T2DM across the country by over 25% in the adult population. By 2030, this rate is expected to double [6,7,8,9,10]. In a recent study, the prevalence of T2DM in Saudi Arabia is 27.6% in females and 34.1% in males [11], with T2DM onset averaging 57.5 years in males and 53.4 years in females [11]. Another study found that in the capital Riyadh, DM prevalence among 30–70 year old Saudis was 23.7% overall, while an additional 14.1% had impaired fasting glucose [12].

In previous studies, lifestyle interventions in individuals with prediabetes were found effective in the prevention or delaying the progression to T2DM [13,14,15,16,17,18]. These studies focused on body weight loss by dietary restrictions and increased physical activities. A previous randomized clinical trial done in prediabetes adults (mean follow-up of 2.8 years) demonstrated that the incidence of T2DM was reduced by 58% through intensive lifestyle modifications [15]. Similarly, in a 3 year follow-up lifestyle intervention from the Finnish Diabetes Prevention study, a 58% reduction in the relative risk to develop T2DM was observed [16]. So far in Saudi Arabia and the Middle East in general, most prediabetes intervention studies were of short duration and none so far have exceeded 1 year of lifestyle modification [19,20]. Hence, whether interest in lifestyle modifications can be sustained beyond one year is yet to be determined in the region. In this 18 month intervention study, we investigated the effects of an 18 month intensive lifestyle modification on prediabetes reversal in Saudi individuals with prediabetes.

## 2. Materials and Methods

### 2.1. Participants

**Figure 1:**
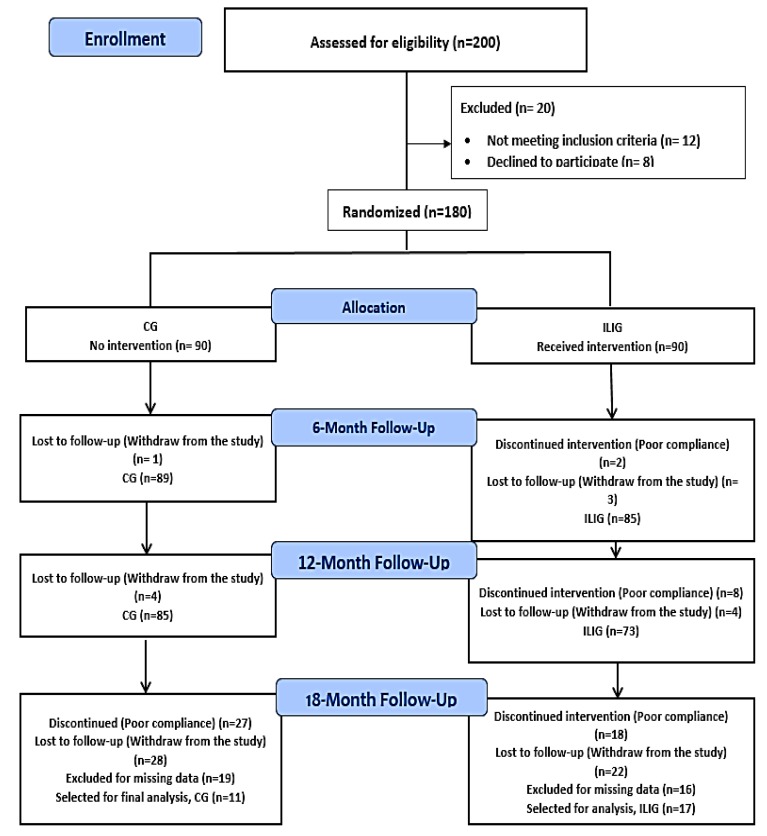
Flow chart of the study participants describing their participation and allocation (CG, control group; ILIG, intensive lifestyle intervention group).

A total of 180 Saudi male and female participants (age range 25–60 years old) were recruited from King Salman Hospital in Riyadh, Saudi Arabia. Before commencement, each participant received informed consent. Inclusion criteria: Overweight or obese individuals (body-mass index (BMI) ≥ 25 kg/m^2^) with impaired fasting serum glucose levels were eligible for the study. Impaired fasting glucose was defined based on the American Diabetes Association (ADA) criteria (serum glucose = 5.6–6.9 mmol/L (100–125 mg/dL) [21]. The use of ADA criteria in the present study, instead of the cut-off proposed by World Health Organization (WHO) (fasting glucose 6.1–69 mmol/L), was to include a bigger number of individuals, given the wider range of glucose level proposed by ADA. Exclusion criteria were: (1) those with T2DM or on T2DM medications; (2) history of malignancy; (3) diagnosed or suspected disease of the liver, pancreas, endocrine organs, or kidney; (4) ischemic heart disease or cerebrovascular disease (or a history of such disease). All study participants had prediabetes at baseline. Using a computer-generated random number list, participants were allocated randomly by block randomization with a 1:1 allocation ratio, to either the intensive lifestyle intervention group (ILIG) or control group (CG). This study was approved by the Ethics Committee, College of Science, King Saud University (KSU); Riyadh, Saudi Arabia (Reference# 8/25/220355). A flow chart describing the study population is provided in Figure 1. The primary outcome in the present study is the reversal of prediabetes to normoglycemia. The secondary outcome is the effect of intervention on blood pressure.

### 2.2. Intervention

Participants were informed individually about T2DM risk factors, its pathogenesis, and the role of dietary restriction and increased physical activity in delaying the onset of T2DM. Participants were advised to modify their lifestyle through shifting to a healthy diet and implementing good exercise behaviors, to increase physical activity and to reduce their body weight. All participants received information about the recommended lifestyle changes in the form of pamphlets and booklets also employed in previous studies [16,22]. In addition, participants were educated every three months through educational sessions about the lifestyle modifications necessary to prevent T2DM. These educational activities took place at the auditoriums of the respective hospitals across both study centers.

The control group (CG) received the normal advice for lifestyle modifications as detailed above. The intensive lifestyle intervention group (ILIG), in addition to the above lifestyle modifications, followed a strict lifestyle modification with individually tailored counseling for improving their diet and exercise behaviors. These strict lifestyle changes suggested were as follows: reducing body weight by at least by 5%, receiving carbohydrates as 50%–60% and fat as less than 30% of their daily energy intake, receiving at least a fiber intake of 15 g/1000 kcal, and lastly, exercising over 150 min/week or 30 min/day at moderate intensity. At each visit with an intervening 6 month interval, the ILIG group had their lifestyle modifications tailored to each participant according to their lifestyle, using a diary, by a registered nutritionist. In addition, ILIG participants were educated about the effect of exercise on the regulation of blood glucose in individuals with prediabetes, and were prescribed aerobic exercise of 30 min five times per week (e.g., bicycling, swimming, badminton, walking, etc.). Based on their health conditions or lifestyle, the frequency, duration, and exercise type were personalized. The CG did not receive lifestyle education sessions, dietary counselling and an on-demand support system. Table 1 shows the differences in interventions given to both groups.

### 2.3. Anthropometric and Biochemical Parameters

Baseline, 6 month, 12 month and 18 month anthropometrics included height (cm), weight (kg), and waist and hip circumferences (cm). Body mass index (BMI) was calculated as weight in kilograms divided by height in square meters, and systolic and diastolic blood pressure were obtained by standard methods. Fasting blood glucose and lipid profile were quantified using routine biochemical tests in an automated biochemistry analyzer (Konelab 20, Thermo-Fischer scientific, Helsinki, Finland). Serum insulin was quantified using multiplex assay kits (LuminexW xMAPW Technology platform) (Luminexcorp, Texas). All laboratory assessments were done at the Chair for Biomarkers of Chronic Diseases (CBCD), College of Science in KSU, Riyadh, Saudi Arabia.

### 2.4. Statistical Analyses

To detect the difference in glucose level in participants, with the effect size of 0.25 at 95%CI and 80% power between the normal and intervention group, the total required sample size is *N* = 130. The effect size used in the present study was made more stringent following a similar study that used an effect size of 0.35 [23]. This study will target a total of 150 patients to compensate loss to follow-up. Descriptive and inferential statistics were performed using SPSS (version 21, IBM). Analysis was done based on per protocol. Data were presented as mean ± standard deviation (SD) for continuous variables, while categorical variables were presented as frequencies. A repeated measures ANOVA was performed to check the intervention and interaction of intervention x time effects. The association between the diabetes status after follow-up and intervention was tested using a chi-square test. The odds ratio was done using generalized estimating equation methods. *p*-value < 0.05 was considered significant.

## 3. Results

A total of 180 (90 in each group) Saudi adults with prediabetes were initially randomized, 158 (85 in CG, 73 in ILIG) at the 12 month follow-up, while 28 participants (11 CG and 17 ILIG) completed the entire 18 months of this intervention. Poor compliance and loss to follow-up were the most common reasons for participants to be excluded from the analyses (Figure 1). At baseline, no significant differences in the study parameters between both groups were found (Table 2).

### 3.1. Anthropometric and Biochemical Characteristics Overtime

At baseline, there were no differences between control and intervention groups in all study parameters (Table 2). After the 12 month intervention, the ILIG mean body weight was significantly lower after 18 months as compared to the baseline (77.7 ± 16.2 vs. 79.6 ± 16.0, *p* < 0.05), while no significant change was observed in CG at 12 months (82.2 ± 13.4 vs. 81.7 ± 13.9). BMI was also significantly reduced in the ILIG post intervention (30.6 ± 6.6 vs. 31.3 ± 6.4, *p* < 0.05), while no significant changes in BMI in the CG were found. Similarly, the waist circumferences significantly decreased overtime in the ILIG post intervention (96.3 ± 13.0 ± vs. 97.9 ± 13.0, *p* < 0.05), but not in the control group. Regarding the diabetes markers, glucose levels significantly decreased in the ILIG post intervention (5.7 ± 0.8 vs. 6.1 ± 0.4, *p* < 0.05) as well as HOMA-IR levels (3.8 ± 0.8 vs. 4.3 ± 0.8, *p* < 0.05) (Table 3). No significant changes were observed in blood pressure in both groups after 18 months.

### 3.2. Primary Outcome: Reversion to Normoglycemia

**Figure 2:**
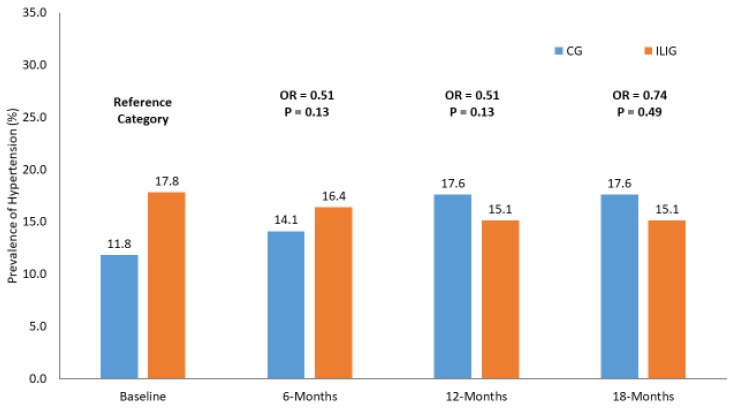
Prevalence of hypertension in both groups over time.

The cumulative incidence of reversion after the 12 month intervention in the ILIG was 38 participants (52.1%) who reverted to normal status, and was significantly better than in the CG, whose 26 participants (30.6%) reverted to normal status after intervention, *p* = 0.02 (Table 4). The incidence of T2DM in the ILIG after 12 months was five participants out of 73 (6.8%) as compared to six participants out of 85 (7.1%) in the CG. For the participants for whom 18 month follow-up data was available, in the control group, out of the 11 patients with prediabetes at the baseline, two developed T2DM, four4 remained at the prediabetes stage while five had normoglycemia. In the ILIG, out of 17 patients with prediabetes at the baseline, five had prediabetes, 12 had normoglycemia, while none developed T2DM. Figure 1 shows the odds ratio (OR) of hypertension in both groups using the baseline as a reference category. No significant differences were seen in the prevalence of hypertension in both groups over time. Worthy to note however is that the odds of hypertension decreased in the ILIG over time, but this change was insignificant (Figure 2).

## 4. Discussion

Individuals with prediabetes are at a high risk for T2DM. Our study aimed to assess lifestyle intervention efficacy on the reversion of prediabetes status to normoglycemia in a cohort of adult Saudi individuals. Intervention was personalized based on the lifestyle of each participant and was planned to reach and preserve the optimum BMI by properly correcting the quantity and composition of meals and increased physical activity. In this study, body weight was significantly decreased, as also did the incidence of diabetes in the ILIG as compared to the CG. In addition, the proportion of participants that reverted to normoglycemia was significantly higher in the ILIG than participants in the CG. In an earlier study on the Japanese population, lifestyle education was significantly effective in reducing the progression rate from prediabetes to diabetes status in a group of participants with lifestyle education intervention at routine health checkups than participants receiving only checkups [24]. The authors suggested that the prevention of the progression from prediabetes to diabetes in the intervention group was due to the decrease of adjustable risk factors, such as body weight, diet, and exercise.

Several previous studies have found that lifestyle intervention in individuals with prediabetes can prevent or delay the progression to T2DM through weight reduction [15,17,25,26,27,28]. Studies of lifestyle interventions targeting weight reduction by a combination of diet and exercise were more efficient in losing visceral fat over subcutaneous fat, which gave rise to a healthier metabolic status [29].

Several epidemiologic studies have suggested that obesity is one of the most significant risk factors for T2DM. Previous prospective studies found that adult obesity has a strong link with T2DM incidence [30,31]. Our results showed that body weight and BMI were significantly reduced in the ILIG, which may have contributed to the reversal of prediabetes progression to diabetes. Additionally, reversion to normoglycemia by lifestyle intervention was seen independently of body weight reduction [28]. Moreover, aside from weight loss, healthy dietary and exercise behaviors were effective components of lifestyle interventions, with the ability to achieve favorable metabolic effects [32,33].

To truly prevent diabetes in individuals with prediabetes, the reversion to normoglycemia is better than just maintaining the prediabetes status. A previous study found that developing diabetes was significantly lower in individuals who returned to normoglycemia than those who consistently maintained a prediabetes condition [34]. This was because of improved insulin sensitivity and pancreatic β-cell function throughout the intervention. Our study showed that the proportion of individuals who became normoglycemic was significantly higher in the ILIG than the controls. Assuming that this reversal is temporary, their risk for T2DM was definitely reduced.

The authors acknowledge several limitations. Dietary intake and physical activity were not actively monitored for participants. Hence, we cannot assess whether the modest weight loss in the ILIG was due to dietary restriction, exercise or both. Second was the high dropout rate in our study, particularly at the 18 month follow-up, therefore restricting the true impact of the intervention in this time duration. Intervention studies are still at an infancy stage in the Middle East in general and are, at present, still building the necessary infrastructure for conducting longer intervention studies to be at par with the more established and set-up research facilities in the West. The large drop out also suggests, but does not prove, that sustaining interest in lifestyle change is difficult to do and that people, particularly the population used in the present study, will revert back to their old ways with time. High attrition rates are unfortunately common for clinical trials done in Saudi Arabia, especially for new interventions. In one study on the 6 month effects of probiotics among Saudi T2DM patients, 150 participants were randomized to receive either placebo or probiotics at baseline, but only 61 completed the trial, as the concept of ingesting live bacteria was relatively unheard of in the region, therefore affecting compliance [35]. In the present study, participants of the intervention were able to successfully apply the lifestyle changes for one year, but the majority declined to continue for 6 months more, citing changes in priorities and other personal reasons that would prevent them from fully engaging in the program. Behavioral techniques such as self-efficacy, goal-setting and provision of feedback should be applied more aggressively in future studies. Harder outcome measures, such as glycated hemoglobin, were not assessed and this could have solidified the efficacy of the intervention. Lastly, the majority of the participants do not have hypertension at baseline, and this may explain the lack of significant changes in blood pressure in both groups over time. Future investigation targeting the hypertensive population may provide further evidence that lifestyle intervention might improve their blood pressure in a non-pharmacological way.

Despite the limitations, the study findings remain robust, and is the longest lifestyle intervention study thus far on diabetes prevention within the Middle East region. The study adds to the current literature and has clinical value, given the ethnic and cultural variations in response to diabetes prevention programs. The challenge of sustaining the interest of people at risk for T2DM in implementing lifestyle changes should be identified and addressed if such programs are to succeed long term within the region.

## 5. Conclusions

Our 18 month lifestyle intervention study demonstrated that reversion from prediabetes to normal status is significantly more effective through a tailored lifestyle intervention than standard care. The reversion to normal status was a direct consequence of improvement in body weight and BMI. The beneficial effects was most pronounced only within 12 months, suggesting, but not implicating, that people at high risk for T2DM within the region find it challenging to sustain lifestyle changes for a longer term. These challenges should be identified and addressed by physicians and primary care givers if we are to successfully combat the rising epidemic of T2DM in the Middle East.

## Figures and Tables

**Table 1:** Differences between study group interventions.

Lifestyle Intervention	CG	ILIG
Baseline Weight reduction (≥5%) Exercise (150 min/week) Reduce fat intake (<30% of total energy) Increase fiber intake (15 g/1000 kcal) diet	Given as a pamphlet and booklet to all participants as a group	Individually explained to each participant by a registered dietitian
Bimonthly lifestyle education sessions every 4 months Dietary counselling Dietary intake record Mode of follow-up On demand support system Blood collection Anthropometrics	No None None As a group No Baseline and every 6 months Baseline and every 6 months	Yes Baseline and every 6 months Baseline and every 6 months Individually Yes Baseline and every 6 months Baseline and every 6 months

**Note**: CG, control group; ILIG, intensive lifestyle intervention group; g, gram; kcal, kilocalories; min, minutes.

**Table 2:** Descriptive statistics according to groups.

Parameters	Control	ILIG	*p*-Values
*N*	85	73	
Age (years)	42.3 ± 11.3	43.4 ± 7.8	0.48
Female/Male	64/21	51/22	0.48
BMI (kg/m^2^)	32.6 ± 5.8	31.3 ± 6.4	0.19
Weight (kg)	81.7 ± 13.9	79.6 ± 16.0	0.17
Waist (cm)	95.6 ± 6.8	97.9 ± 13.0	0.38
WHR	0.9 ± 0.0	0.9 ± 0.1	0.60
Systolic BP (mmHg)	120.0 ± 12.1	122.1 ± 15.8	0.36
Diastolic BP (mmHg)	76.4 ± 9.7	76.0 ± 11.9	0.87
Glucose (mmol/L)	6.0 ± 0.4	6.1 ± 0.4	0.18
Insulin (uU/mL)	15.1 ± 5.0	15.9 ± 2.6	0.24
HOMA-IR	4.1 ± 1.4	4.3 ± 0.8	0.27

**Note**: Data presented as mean ± SD for normal variables while median (1st–3rd quartile) for non-normal variables; *p* < 0.05 is considered significant.

**Table 3:** Descriptive statistics according to groups and time.

Parameters	Control (*N* = 85)			Intervention (*N* = 73)			Between Groups
	Baseline (*N* = 85)	6 Months (*N* = 85)	12 Months (*N* = 85)	Baseline (*N* = 73)	6 Months (*N* = 73)	12 Months (*N* = 73)	*p*-Values
Age (years)	42.3 ± 11.3			43.4 ± 7.8			--
Female/Male	64/21			51/22			--
BMI (kg/m^2^)	32.6 ± 5.8	32.8 ± 5.9	32.8 ± 5.7	31.3 ± 6.4	31.0 ± 6.7	30.6 ± 6.6 ^AB^	0.07
Weight (kg)	81.7 ± 13.9	82.3 ± 13.9	82.2 ± 13.4	79.6 ± 16.0	78.7 ± 15.9	77.7 ± 16.2 ^AB^	0.15
Waist (cm)	95.6 ± 6.8	95.7 ± 6.7	95.5 ± 6.2	97.9 ± 13.0	97.7 ± 13.5	96.3 ± 13.0 ^AB^	0.28
WHR	0.9 ± 0.0	0.9 ± 0.1	0.9 ± 0.1	0.9 ± 0.1	0.9 ± 0.1	0.9 ± 0.1	0.41
Systolic BP (mmHg)	120.0 ± 12.1	118.8 ± 13.4	119.2 ± 15.8	122.1 ± 15.8	120.9 ± 16.8	119.5 ± 16.6	0.50
Diastolic BP (mmHg)	76.4 ± 9.7	75.4 ± 9.1	77.1 ± 13.7	76.0 ± 11.9	75.1 ± 10.8	75.9 ± 12.8	050
Glucose (mmol/L)	6.0 ± 0.4	6.1 ± 0.7	5.9 ± 0.9	6.1 ± 0.4	5.7 ± 0.8 ^A^	5.7 ± 0.8 ^A^	0.03
Insulin (uU/mL)	15.1 ± 5.0	15.1 ± 6.1	15.2 ± 6.0	15.9 ± 2.6	15.3 ± 2.9	15.1 ± 2.5	0.67
HOMA-IR	4.1 ± 1.4	4.1 ± 1.6	4.0 ± 1.6	4.3 ± 0.8	3.8 ± 0.8 ^A^	3.8 ± 0.8 ^A^	0.66

**Note**: Data presented as mean ± SD; superscripts A and B indicate significance from the baseline and 6 months (within group interaction effects) respectively, *p*-values are obtained from a repeated measures ANOVA; *p* < 0.05 is considered significant.

**Table 4:** T2DM of participants in different time points.

	6 Months			12 Months			18 Months		
	CG	ILIG	*p*-Value	CG	ILIG	*p*-Value	CG	ILIG	*p*-Value
	N (%)	N (%)		N (%)	N (%)		N (%)	N (%)	
T2DM	6 (7.1)	6 (8.2)	0.04	6 (7.1)	5 (6.8)	0.02	2 (18.2)	0 (0.0)	0.18
Pre-DM	66 (77.6)	44 (60.3)		53 (62.4)	30 (41.1)		4 (36.4)	5 (29.4)	
Normal	13 (15.3)	23 (31.5)		26 (30.6)	38 (52.1)		5 (45.5)	12 (70.6)	

**Note**: *P*-values were obtained from chi-square and exact test; *p* < 0.05 is considered significant.
